# Identification of 2,4-Di-*tert*-Butylphenol as an Antimicrobial Agent Against *Cutibacterium acnes* Bacteria from Rwandan Propolis

**DOI:** 10.3390/antibiotics13111080

**Published:** 2024-11-13

**Authors:** Florent Rouvier, Lydia Abou, Emmanuel Wafo, Perrine Andre, Julien Cheyrol, Mohamed-Mohsen Khacef, Claude Nappez, Hubert Lepidi, Jean Michel Brunel

**Affiliations:** 1 Aix Marseille Université, INSERM, SSA, MCT, 13385 Marseille, France; rouv.flo@hotmail.fr (F.R.); emmanuel.wafo@univ-amu.fr (E.W.); 2 Observatoire Français d’Apidologie (OFA), La Garniere, Route de Mazaugues, 83136 Mazaugues, France; 3 C2VN, Aix Marseille Université, INSERM 1263, INRAE 1260, 13385 Marseille, France; lydia.abou@univ-amu.fr; 4 CEFOS, UAR2020, Aix Marseille Université, Campus Timone, 27 Boulevard Jean Moulin, 13005 Marseille, France; perrine.andre@univ-amu.fr (P.A.); julien.cheyrol@univ-amu.fr (J.C.); mohamed-mohsen.khacef@univ-amu.fr (M.-M.K.); claude.nappez@univ-amu.fr (C.N.); 5 Laboratoire d’Anatomie Pathologique, Hôpital de la Timone, 13005 Marseille, France; hubert.lepidi@ap-hm.fr

**Keywords:** acne, antibacterial activity, *Cutibacterium acnes*, 2,4-Di-*tert*-butylphenol, ointment, propolis

## Abstract

Background/Objectives: Acne is the most prevalent dermatological condition among humans, affecting approximately 80% of adolescents during puberty. To date, numerous compounds have been used for acne treatment, including erythromycin ointments and antiseptics, with varying degrees of success. The emergence of erythromycin-resistant *C. acnes* strains has spurred the search for new antimicrobial agents, particularly from natural sources. Methods: Propolis collected in Rwanda was extracted and fractionated by flash chromatography and tested against *C. acnes* growth by using NCLSI recommendations. Results: In our research, we identified a molecule, 2,4-Di-*tert*-butylphenol (2,4-DTBP) which inhivbited the *C. acnes* growth at a concentration of 16 µg/mL. Based on these results, we formulated an ointment (1%) using OFAP18 and petroleum jelly for the potential treatment of acne using a mouse model. Conclusions: In vitro and in vivo evidence suggests that 2,4-DTBP has anti-inflammatory properties and could effectively manage the overgrowth of *C. acnes* as well as serve as a potent alternative for the formulation of an active propolis ointment for acne treatment.

## 1. Introduction

Acne stands out as the prevailing dermatological condition among humans, primarily affecting approximately 80% of adolescents during the onset of puberty, a period marked by hormonal fluctuations that are uniform across genders. While acne typically diminishes as individuals enter their twenties, it persists in 54% of women and 40% of men in adulthood. The development of acne is closely linked to the distribution of sebaceous glands, primarily appearing on the face, jawline (especially in adults), neck, chest, and back [1]. It presents in various forms, including whiteheads, blackheads, and differing levels of inflamed lesions [2]. Antibiotics play a pivotal role in restraining bacterial growth, with oral antibiotics serving as the primary treatment for moderate acne or instances where topical combinations prove intolerable or ineffective. Notably, systemic erythromycin and various generations of tetracyclines have demonstrated efficacy in addressing inflammatory acne. However, the prolonged use of topical and oral antibiotics against *C. acnes* has led to a prevalent emergence of resistance, particularly in European Union countries [3,4,5]. Spanish researchers, for instance, reported resistance rates of 91% and 92.4% for both kinds of antibiotics in Spain, while Greece and Italy recorded resistance rates of 75.3% and 59.5%, respectively [6]. The escalating prevalence of drug-resistant *C. acnes* strains globally has prompted widespread concern about the diminishing arsenal of antibiotics available for treating this common ailment [7,8,9].

Propolis, a resinous substance crafted by honeybees, boasts a rich history in traditional medicine spanning millennia. Etymologically rooted in ancient Greek, the term “propolis” combines ‘pro’ (in front of) and ‘polis’ (city), literally translating to ‘defense of the city’ [10]. This aptly reflects how bees utilize propolis to safeguard their hive from diseases caused by fungi, bacteria, and predators [11]. Derived from tree buds and bark, propolis, with its sticky and gummy consistency, results from the collection of resins by foraging bees, creating a blend with pollen, waxes, and enzymes [12,13]. The exact composition varies based on honeybee species, plant source, harvesting seasons, and climate changes. Propolis has demonstrated a spectrum of clinical applications, including antioxidant, anticancer, anti-inflammatory, and antimicrobial effects [14,15,16,17,18,19,20,21]. Notably, recent findings highlight propolis as a natural remedy for skin issues, thanks to its soothing and healing properties [22].

Nonetheless, it is crucial to recognize that propolis is not a singular entity; rather, its variations depend on the surrounding flora, and not all types will necessarily exhibit the same efficacy in addressing a particular pathology.

In the pursuit of discovering potent antimicrobial molecules, our studies have delved into exploring the potentialities of different propolis samples collected in Rwanda, a region marked by the presence of one of the last remaining primary forests in the world. Our aim is to identify novel molecules of interest to advance antimicrobial research, specifically for developing a potent alternative for formulating an active propolis-based ointment for acne treatment.

## 2. Materials and Methods

### 2.1. Reagents

All solvents and reagents used were commercially available. Dichloromethane, methanol, ethyl acetate, and petroleum ether were purchased from VWR and used without further purification. Column chromatography was performed on Macherey Nagel silica gel (70–230 mesh) using a COMBIFLASH device. The ^1^H and ^13^C NMR spectra were recorded in CDCl_3_ on a 300 and 75 MHz Bruker AC 300 spectrometer, respectively (the usual abbreviations are used: s: singlet, d: doublet, t: triplet, q: quadruplet, m: multiplet). All chemical displacements are indicated in ppm. The analysis by mass spectroscopy was carried out by the laboratory of analysis of the faculty of pharmacy (University of Aix-Marseille).

### 2.2. Preparation of the Hydroalcoholic Extract of OFAP18

OFAP18 was collected in Rwanda at a precise place located at the GPS coordinates 2°26′53.0″ S 29°04′07.0″ E, in the form of a black gum. Briefly, 9.0 g of propolis OFAP18 was extracted with 15 mL of ethanol (water–ethanol (30:70, *v*/*v*)) by using a Biotage initiator microwave apparatus operating for 10 min at 100 °C and 2 bars. After centrifugation and filtration, this led to an ethanol extract of which the propolis concentration was determined to be 120 mg/mL and was subsequently used for biological tests and flash chromatography (from non-polar to polar, namely petroleum ether, ethyl acetate, and methanol).

### 2.3. Fractionation of Propolis OFAP18

Briefly, 9.0 g of propolis OFAP18 was extracted with ethanol (water–ethanol (30:70, *v*/*v*) leading to an ethanol extract which was concentrated under vacuum to a crude powder (1.8 g) and subsequently submitted for flash chromatography (from non-polar to polar namely petroleum ether, ethyl acetate, and methanol) yielding 73 fractions of 20 mL which were grouped into 11 fractions titled F1 to F11

### 2.4. Bacterial Strains

The bacterial strains tested were *E. coli* ATCC25922, *B. cereus* ATCC11778, *S. aureus* ATCC25923, *S. epidermidis* CIP81.55, and *C. acnes* DSM1897, CIP110516, CIP110517, CIP110528, and CIPA179. All bacterial experiments were performed according to *CLSI M11 Methods for Antimicrobial Susceptibility Testing of Anaerobic Bacteria, 9th Edition* and *CLSI M07_Methods for Dilution Antimicrobial Susceptibility Tests for Bacteria That Grow Aerobically, 12th Edition*.

### 2.5. General Procedure for Measuring Antimicrobial Activities

The antibacterial activity of the compounds was measured using a standard microdilution test based on Clinical and Laboratory Standards Institute (CLSI) guidelines. This method was slightly modified to improve reproducibility. The final volume remains 200 µL, now consisting of 190 µL of bacterial suspension and 10 µL of the test molecule solution.

The bacteria were handled under a hood in the L2 laboratory. The chemical compounds (2,4-Di-*tert*-butylphenol, 3,5-Di-*tert*-butylphenol, 2,6-Di-*tert*-butylphenol) and propolis extracts to be tested were prepared in 70% ethanol at a concentration of 10 mg/mL.

#### 2.5.1. Culture Preparation

An inoculum was prepared by resuspending a grown colony (1 to 7 days) of each strain in a culture tube containing 5 mL of Brain Heart Infusion (BHI) medium then incubated at 37 °C for 16 to 18 h under agitation at 100 rpm. Similar experimental conditions are involved for *C. acnes* but under an anaerobic atmosphere (GENbag anaer—Biomérieux^®^, Paris, France) and during a 30 to 36 h incubation.

#### 2.5.2. Preparation of Precultures

Each laboratory strain has an established OD-to-CFU ratio. To determine this ratio, all strains were cultured until reaching the exponential growth phase. The optical density (OD) of each bacterial suspension was measured, followed by bacterial enumeration on solid media. This ratio enables the precise calculation of the required dilution, ensuring the appropriate bacterial concentration for Minimum Inhibitory Concentration (MIC) tests. All the experiments were performed according to CLSI guidelines. Typically, the precultures were prepared by adding 20 µL of overnight *E. coli* ATCC25922 culture, 50 µL of *B. cereus* ATCC11778, or 100 µL of *S. aureus* ATCC25923 and *S. epidermidis* CIP81.55, each into 3 mL of fresh BHI. The tubes were then incubated at 37 °C for 3–4 h at 100 rpm. Due to the slower growth rate of *C. acnes*, a previously prepared culture was used directly for microplate preparation.

#### 2.5.3. Preparation of Test Solutions

The diluted molecules (2,4-DTBP, 2,6-DTPB, and 3,5-DTPB) (in 70% ethanol) or the hydroalcoholic extract of propolis samples (OFAP2-21) underwent a two-fold serial dilution, beginning with 70% ethanol for the first two dilutions, followed by dilution in water. Working solutions with concentrations ranging from 5000 to 80 µg/mL (corresponding to ethanol percentages of 70% to 2.2%) were prepared, resulting in final concentrations in the microplate wells from 250 to 4 µg/mL (3.5% to 0.1% ethanol).

#### 2.5.4. Preparation of Microplate for Determination of MIC

The optical density at 600 nm (OD600) of each bacterial suspension was measured and then diluted to achieve a target OD600 (0.0009 for *E. coli*, 0.001 for *B. cereus*, 0.0012 for *S. aureus* and *S. epidermidis*, and 0.0013 for *C. acnes*), corresponding to a bacterial density of 5 × 10^5^ CFU/mL as previously determined in our laboratory. For example, an OD_600_ of 0.5 corresponds to a concentration of 2.1 × 10^8^ CFU/mL for *S. aureus*. Therefore, if this suspension is diluted 417-fold to an equivalent OD of 0.0012 (used solely for calculation purposes and not directly measured), a final bacterial concentration of 5 × 10^5^ CFU/mL is obtained. In a flat-bottom, translucent 96-well plate, 10 µL of each working solution—within the dilution range of propolis extract or tested molecules (2,4-DTBP, 2,6-DTPB, and 3,5-DTPB)—was added to 190 µL of the bacterial suspension, adjusted to 5 × 10^5^ CFU/mL. This resulted in final concentration ranges of 250 to 4 µg/mL, with final ethanol concentrations ranging from 3.2% to 0.1%

The control wells were carried out with the following parameters:

- Growth control containing 200 µL bacterial suspension at 5.10^5^ CFU/mL.
- Contamination control containing 200 µL of medium BHI.
- Control growth in the presence of ethanol, corresponding to a range of ethanol between 3.2 and 0.1% final in the presence of bacterial suspension at 5.10^5^ CFU/mL.

#### 2.5.5. Reading the Plates

After an 18-hour incubation at 37 °C for *E. coli* ATCC25922, *B. cereus* ATCC11778, *S. aureus* ATCC25923, and *S. epidermidis* CIP81.55, or a 40-hour anaerobic incubation at 37 °C for *C. acnes* strains DSM1897, DSM30753, CIP110516, CIP110517, CIP110528, and CIP.A179, 50 µL of a 2 mg/mL nitrophenyl-tetrazolium iodide (INT) solution was added to each test well. In the presence of live bacteria, the INT is reduced, producing a detectable red compound.

Growth and ethanol control wells must indicate bacterial growth, while contamination control wells must show no growth. Once these conditions are confirmed, the test wells can be interpreted.

For each product tested, the minimum inhibitory concentration (MIC) is defined as the lowest concentration of the test product that does not result in a visible red color change, indicating bacterial inhibition.

### 2.6. Growth Curves

The solutions of the compounds at the tested concentrations of 2, 4, 16, and 32 μg/mL were each tested in triplicate against *B. cereus* ATCC11778. In a 96-well plate, 10 μL of 40, 80, 320, and 640 μg/mL fresh stock solutions of 2,4-DTBP, 2,6-DTPB, and 3,5-DTPB compounds were placed, as well as 190 μL of 5 × 10^5^ CFU/mL of the selected bacterial suspension in brain heart infusion (BHI) broth.

Positive controls containing only 200 μL of 5 × 10^5^ CFU/mL of the bacterial suspension in BHI and negative controls containing only 200 μL of BHI broth were added. The plate was incubated at 37 °C in a TECAN Spark Reader (Roche Diagnostic, Meylan, France) and the bacterial growth was followed by OD590 nm measurements every 20 min for 18 h.

### 2.7. Measurement of the ATP Efflux

Fresh stock solutions of 2,4-DTBP, 2,6-DTPB, and 3,5-DTPB were prepared in Phosphate-Buffer Saline (PBS) at concentration of 10 mg/mL then 10-fold diluted in BHI.

The *B. cereus* ATCC11778 suspensions were prepared in BHI and were incubated at 37 °C until reaching the growth exponential phase. Then, 90 μL of bacterial suspension was added to 10 μL of the compound solution in a Corning^®^ 96-well white flat-bottom cell culture plate and shaken for 5s in the incubator at 37 °C. Subsequently, after 3 min of contact, 50 μL of Luceferin–Luciferase reagent (Yelen, Marseille, France) was added to the mixture, and the luminescent signal was quantified with TECAN Spark Reader (Roche Diagnostic, Meylan, France) (Tecan, Männedorf, Switzerland) for 6 readings spaced 30 s apart. Squalamine (100 μg/mL) was used as the positive control to quantify the maximum level of ATP efflux with BHI as the negative control. This assay was performed in three independent experiments.

### 2.8. OFAP18 and Erythromycin Ointments Preparation

The propolis OFAP18-based ointment (1%) was prepared by mixing 95 g petroleum jelly (Cooper, cooperation pharmaceutique française, Paris, France) and 5 mL of a glycerol solution containing 1 g of pure extract of OFAP 18. Thus, petroleum jelly was added gradually, and the contents were mixed for 10 min until obtaining a homogeneous ointment. The ointment was then stored in a sterile tube at 4 °C. A similar protocol was followed to prepare a 1% erythromycin ointment by dissolving 1 g of erythromycin in 4 g of glycerol, then mixing it with 95 g of petroleum jelly. The sterility of the different ointments was then verified by suspending them in sterile distilled water and depositing 100 µL of these suspensions on TSA agar plates and incubating them at 37 °C for 24 h.

### 2.9. Mouse Model of C. acnes Induced Inflammation (APAFIS Project Authorization No. 40597-2023020110385822 v2)

Propolis solutions at 1% (OFAP 18) and erythromycin at 1% in petroleum jelly are used for treatments. Pure petroleum jelly was used as a negative control. Erythromycin (Sigma, Saint Quentin Fallavier, France, ref E7904-10G), the predominant antibiotic used to treat acne, will serve as the reference treatment in this trial.

After a 12-day acclimatization period, the mice received the following cream applications:

- Cohort 1 “test”: 10 mice (5 males and 5 females) received a daily application of petroleum jelly on their left flank and petroleum jelly + 1% propolis on their right flank for 8 days.
- Cohort 2 “reference”: 10 mice (5 males and 5 females) received a daily application of petroleum jelly on their left flank and petroleum jelly + 1% erythromycin on their right flank for 8 days.

On the 9th day, a bacterial suspension of *C. acnes* DSM 1897 at 10^9^ CFU/mL was prepared from overnight cultures in an anaerobic atmosphere following this protocol:

For 24 tubes:

Centrifuge 1.5 mL of culture at 4000 RPM for 10 min.

- Repeat centrifugation twice, removing the supernatant and adding 1 mL of sterile PBS each time, followed by another centrifugation at 4000 RPM for 10 min.
- Remove the supernatant and add 1 mL of sterile PBS.
- Mix and transfer the suspension sequentially from tube 1 to tube 24, each time adding 1 mL of sterile PBS.
- Perform a final centrifugation at 4000 RPM for 10 min and remove the supernatant, then add 1 mL of sterile PBS.

The OD600 of the sample was measured at 2.6. Due to the saturation limit of the device, a 5-fold dilution was prepared for measurement to confirm the bacterial concentration range (1.5 × 10^9^ CFU/mL). Two bacterial counts were conducted on this suspension: the first before starting the injections (1.1 × 10^9^ CFU/mL) and the second 2 h after the injections (1.4 × 10^9^ CFU/mL). These results indicate no significant difference in the quantity of bacteria injected between the first and last treated mice.

The 20 mice were infected by subcutaneous injections of 20 µL of this bacterial suspension. Each mouse received one injection in the right flank and one in the left flank. Following the injection and in the subsequent days, the mice received cream applications according to the previous protocol.

One day after the appearance of pimples, the mice were sacrificed. A 5 mm skin sample from each flank was taken using a punch. Each sample was divided into two portions: one for bacterial enumeration and the other for histological analysis.

### 2.10. Histological Analysis

For each mouse, skin biopsy obtained from the acne pimples treated with petroleum jelly, erythromycin or OFAP18 was fixed in 4% phosphate-buffered neutral formaldehyde and processed for paraffin embedding. Paraffin sections (3–4 μm) were stained with routine hematoxylin–eosin–saffron. The examination was performed by a pathologist blinded to the group identity (H. L.) to determine the level of inflammation of the different skin samples.

## 3. Results and Discussion

In the extraction process, it is noteworthy that propolis exhibits a hard and crumbly consistency initially. However, upon handling and slight heating, it undergoes a transformation, becoming viscous and sticky, with a melting point at temperatures around 70 °C [8]. Among all the propolis tested and collected from various parts of Rwanda, even close in terms of distance, only one, namely OFAP18, demonstrated a potent interesting antimicrobial activity against *C. acnes*, as illustrated in Table 1. Based on these findings, we decided to identify the molecule or molecules responsible for such antibacterial activities.

**Figure 1:**
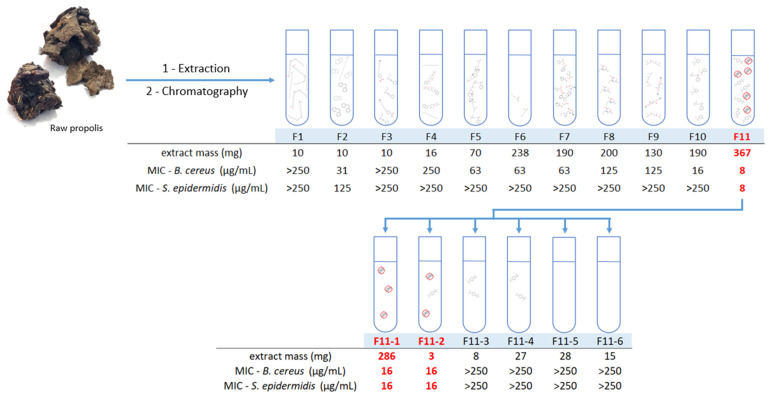
Summary of the fractionation of OFAP18.

Briefly, 9.0 g of propolis OFAP18 was extracted with ethanol (water–ethanol (30:70, *v*/*v*), leading to an ethanol extract which is concentrated under vacuum as a crude powder and subsequently submitted to flash chromatography (from non-polar to polar, namely petroleum ether, ethyl acetate, and methanol) yielding 11 fractions titled F1 to F11 (Figure 1).

**Figure 2:**
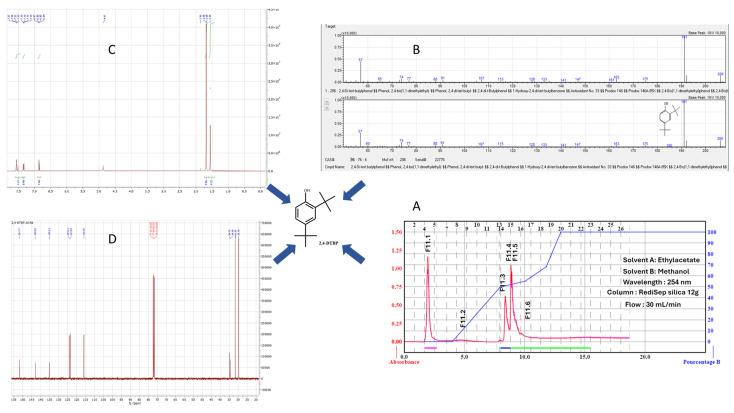
(**A**–**D**) Determination of the identity of 2,4-Di-tert-butylphenol (2,4-DTBP) in the studied fraction.

All the fractions were tested for antimicrobial activities and only fraction F11 led to an interesting MIC against both *B. cereus* and *C. acnes* strains. Thus, the F11 fraction was used for a second purification via flash chromatography carried out from 367 mg of crude residue which allowed us to obtain 35 fractions of 20 mL; these were grouped into six fractions coded from F11.1 to F11.6. These fractions are summarized in Table 2, and their respective antibacterial activities are highlighted. Pure fraction F11.1 was identified as the most active against both *B. cereus* and *C. acnes*, prompting an investigation into the compound responsible for this activity. The nature of the molecule was clearly determined by GC-MS as 2,4-Di-*tert*-butylphenol (2,4-DTBP) and its structure was unambiguously confirmed by ^1^H and ^13^C NMR spectrometry (Figure 2 and Figures S1 and S2).

Notably, we also detected the presence of 3,5-Di-*tert*-butylphenol (3,5-DTBP), an isomer of 2,4-DTPB in a 1:10 ratio relative to 2,4-Di-*tert*-butylphenol, in the analyzed propolis sample via GC-MS analysis.

**Figure 3:**
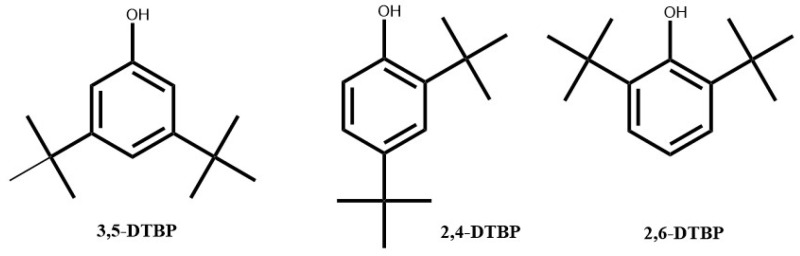
Structure of Di-*tert*-butyl phenol isomers (2,4-DTBP, 3,5-DTBP, and 2,6-DTBP).

All these compounds as well as their 2,6-DTBP isomer (Figure 3) were tested for their intrinsic antimicrobial activities against a wide range of bacterial strains, including *E. coli* ATCC 25922, *B. cereus* ATCC 11778, *S. aureus* ATCC 25923, *S. epidermidis* CIP81.55, and *C. acnes* ATCC 1897.

The antimicrobial activity tests of the commercial molecules 2,4-DTBP and 3,5-DTBP demonstrated strong antimicrobial activity against *B. cereus* and *C. acnes*, particularly with 2,4-DTBP, whereas no activity was encountered by using isomer 2,6-DTBP. Thus, there is a strong correlation between the antimicrobial activity of the OFAP18 extract against the pathogen responsible for acne and the presence of 2,4-DTBP, a property that has not been previously reported in the literature (Table 2).

Subsequently, we were able to extend the spectrum of action of 2,4-DTBP on a larger panel of *C. acnes* strains, confirming the great activity of the latter towards this pathogen with a MIC of 16 µg/mL even against an erythromycin-resistant strain (Table 3).

**Figure 4:**
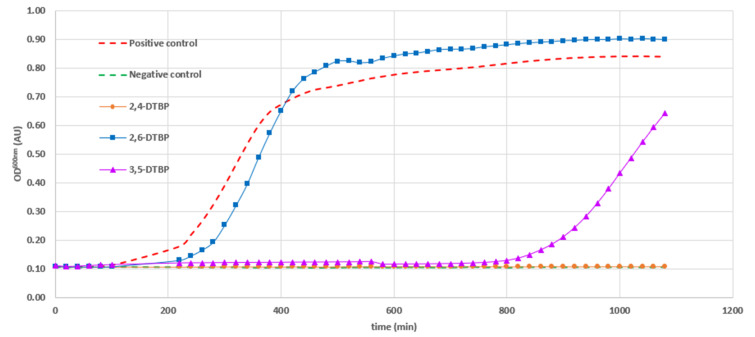
Bacterial growth inhibition against *B. cereus* ATCC 11778 exhibited by Di-*tert*-butyl phenol isomers (2,4-DTBP, 3,5-DTBP, and 2,6-DTBP) used at a 16 µg/mL concentration. Positive control was bacteria only and negative control was media only.

The growth inhibition profiles of the three Di-*tert*-butylphenol (DTBP) isomers against *Bacillus cereus* ATCC 11778 were determined to evaluate the dynamics of their antibacterial activity. No inhibition of bacterial growth was observed in the presence of 2,6-DTBP at a concentration of 16 µg/mL. In contrast, the growth response to 2,5-DTBP was more nuanced; inhibition was observed during the first 13 h, followed by a subsequent escape from the action of the compound. Finally, significant growth inhibition was only observed with the 2,4-DTBP isomer used at a 16 µg/mL concentration (Figure 4).

**Figure 5:**
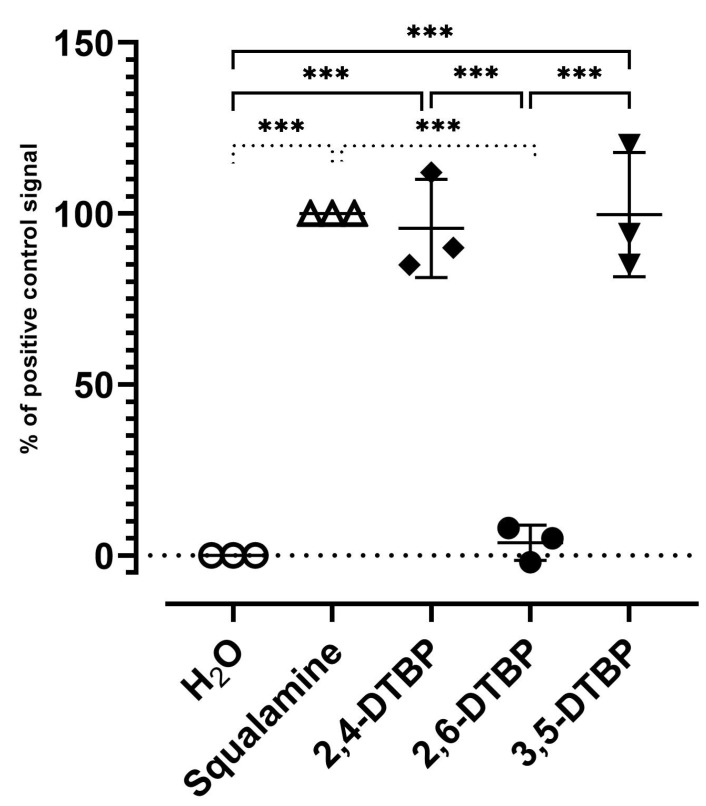
ATP release in *B. cereus* ATCC11778 exhibited by Di-tert-butyl phenol isomers (2,4-DTBP, 3,5-DTBP and 2,6-DTBP) as determined using ATP efflux assay. Squalamine (100 µg/mL) was the positive control and water was the negative control. Compounds were tested at a final concentration of 100 µg/mL, and the results are reported as a percentage (%) relative to positive control. *** shows significant differences.

On the other hand, we attempted to elucidate more precisely the mechanism of action of 2,4-DTBP by investigating its potent permeabilizing and disrupting behavior of the outer membrane of Gram-positive *B. cereus*. A bioluminescence method was then developed involving the detection of the external concentration of ATP, which was used as a reporter reflecting the permeabilizing effect of 2,4-DTBP and 3,5-DTBP. Thus, these derivatives dramatically disrupted the *B. cereus* membrane after 2 min, as observed by intracellular ATP release kinetics, which was like the positive control squalamine (Figure 5). Nevertheless, we must keep in mind that the concentration used for these compounds was very high, whereas only 2,4-DTBP was active at around 16 µg/mL, suggesting that LPS damage induced by this latter is clearly greater and faster than that caused by 2,6-DTBP. Conversely, no significant effect was found by using water as a negative control as well as 2,6-DTBP during the test time, with only a 0 and 10% ATP efflux release relative to the squalamine positive control, respectively.

Based on these results, we formulated an ointment using OFAP18 and petroleum jelly (1% OFAP18 or erythromycin) for the treatment of acne in a mouse model. In the first set of experiments, we injected 20 µL of a bacterial suspension (10^9^ CFU/mL) under the skin to induce acne pimples over 2 days, followed by applying the ointment for 7 days. We mainly observed a decrease in pimples for each test parameter. No bacteria were enumerated, and all mice exhibited inflammation; the immune response of mice masked any potential effect of the formulated ointment.

**Figure 6:**
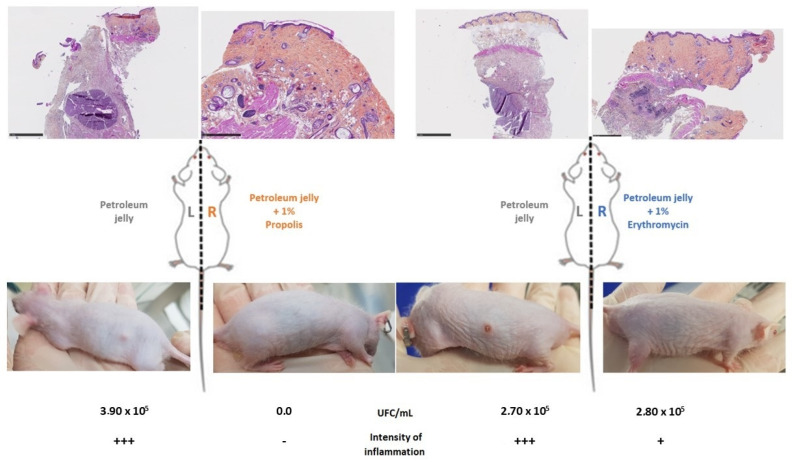
OFAP18 (orange)- and erythromycin (blue)-treated mice exhibited lower epidermal inflammation than control ones treated with excipient 2 days after injection with 10^9^ CFU/mL of *C. acnes* DSM1897 suspension.

In the second set of experiments, we applied OFAP18 and erythromycin ointments daily for 8 days before injecting the bacterial suspension. We then evaluated the efficacy of both ointments against *C. acnes* colonization over the course of 2 days (Figure 6). The histological analysis of mouse skin biopsies showed inflammatory infiltrates in the dermis composed predominantly of neutrophils often associated with cutaneous necrosis. OFAP18 and erythromycin-treated mice exhibited lower dermal inflammation than control ones treated with excipient 2 days after injection with 10^9^ CFU/mL of *C. acnes* DSM1897 suspension.

**Figure 7:**
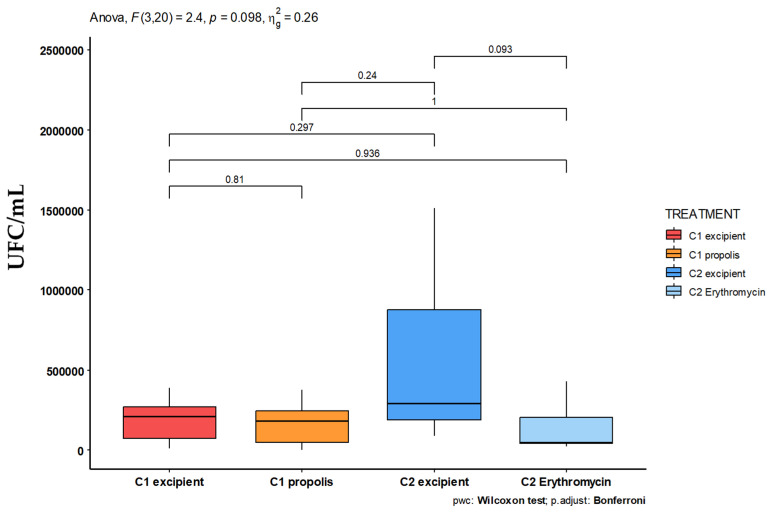
Graphical representation of bacterial counts of *C. acnes* based on the treatments performed on the two cohorts of mice with OFAP18 (C1 orange) or erythromycin (C2 pale blue) ointments.

Moreover, due to the lower observed concentration of bacteria in the petroleum sample from the cohort 1 mice compared to cohort 2, pre-treatment with the 1% OFAP18 ointment appears to diffuse throughout the entire body of the mouse, effectively limiting the growth of *C. acnes* bacteria compared to pre-treatment with the petroleum jelly of cohort 2 (Figure 7).

Therefore, due to significant variations in the number of bacteria counted within the mouse categories, we did not achieve statistically significant results regarding the reduction in the bacterial population. However, the comparison between pimples treated with OFAP18 (cohort 1) and those treated with petroleum jelly alone (cohort 2, without propolis diffusion) reveals a significant decrease in *C. acnes* concentration.

Finally, mice treated with OFAP18 and erythromycin exhibited significantly lower epidermal inflammation compared to those treated with petroleum jelly (Figure 7). Notably, more prominent inflammation was observed in mice pre-treated with petroleum jelly than in those pre-treated with OFAP18. We observe a statistically significant difference in bacterial counts between the petroleum jelly and erythromycin treatments in cohort 2 mice (*p* = 0.093). However, no such difference is seen between the OFAP18 treatment and petroleum jelly alone. Notably, there is a significant difference in the bacterial counts of pimples treated with petroleum jelly between cohorts 1 and 2. Furthermore, the pimples in cohort 2 appear more pronounced than those in cohort 1, suggesting that the active compound in propolis may have diffused during the 7-day pre-treatment period. When comparing the bacterial counts of pimples treated with OFAP18 in cohort 1 to those treated with petroleum jelly alone in cohort 2, we observe a marked difference, highlighting the antibacterial effect of the propolis OFAP18 treatment. These data support the effectiveness of the propolis-based cream OFAP18 and, more broadly, the molecule 2,4-DTPB responsible for its antibacterial activity. On the other hand, 2,4-DTPB appears as a metabolite produced by various plants and, even if the biosources and bioactivities have been well investigated, the phenol has not been systematically reported. Thus, it can be, for example, encountered in *Pinus kesiya* as the major component in the water extracts of fresh needles (16%) [23,24,25]. Based on the GPS coordinates (2°26′53.0″ S 29°04′07.0″ E), we were able to determine that two main species could constitute the source of 2,4-DTBP. Indeed, the hives from which the propolis OFAP18 is extracted from are found in a forest of *Eucalyptus globulus* bordered by a green tea field but also by the primary forest of Nyungwe which holds many endemic species of Rwanda.

## 4. Conclusions

In summary, we identified the molecule 2,4-Di-tert-butylphenol (2,4-DTBP) from propolis OFAP18 collected in Rwanda, which demonstrated the inhibition of *C. acnes* growth at a concentration of 16 µg/mL. A 1% ointment formulation was successfully prepared and tested in a mouse model, suggesting that 2,4-DTBP could effectively manage *C. acnes* overgrowth and serve as a potent alternative for acne treatment. Studies are now under current investigation to more precisely identify the most suitable propolis collection site and estimate the quality of the propolis harvested from the seasonality.

## Figures and Tables

**Table 1:** Antimicrobial activities of Rwandan propolis extracts against various both Gram-positive and negative bacteria.

Sample	MIC (µg/mL)						
	*E. coli* ATCC 25922	*S. aureus* ATCC 25923	*S. epidermidis* CIP 81.55	*B. cereus* ATCC 11778	*C. acnes* DSM 1897	*C. acnes* DSM 30753	*C. acnes* CIP 110516
OFAP 2	>256	>256	256	16	8	16	32
OFAP 6	>256	>256	>256	125	125	64	256
OFAP 7	>256	>256	>256	64	64	32	32
OFAP 9	>256	>256	256	32	32	16	ND
OFAP 10	>256	>256	32	64	16	32	ND
OFAP 11	>256	>256	>256	64	64	64	ND
OFAP 15	>256	64	32	16	16	ND	ND
OFAP 16	>256	>256	>256	32	32	ND	ND
OFAP 18	>256	64	16	16	16	16	16
OFAP 20	>256	>256	>256	64	16	32	>256
OFAP 21	>256	64	64	32	32	32	>256

**Table 2:** Antimicrobial activities of OFAP18, 2,4-DTBP, 3,5-DTBP, and 2,6-DTBP against a wide range of bacterial strains.

Sample	MIC (µg/mL)				
	*E. coli* 25922	*S. aureus* 25923	*S. epidermidis* 81.55	*B. cereus* 11778	*C. acnes* 1897
OFAP18	>256	64	16	16	16
2,4-DTBP	>256	16	16	8	16
2,6-DTBP	>256	>256	125	256	>256
3,5-DTBP	>256	32	32	16	64

**Table 3:** Antimicrobial activities of 2,4-DTBP against a large panel of *C. acnes* bacterial strains.

Sample	CMI (µg/mL)					
	*C. acnes* DSM1897	*C. acnes* CIPDSM110512	*C. acnes* CIP 110516 (ERY-R)	*C. acnes* CIP110517	*C. acnes* CIP110528	*C. acnes* CIP A179
2,4-DTBP	16	16	16	16–32	16	16

## Data Availability

The original contributions presented in the study are included in the article/Supplementary Material, further inquiries can be directed to the corresponding author/s.

## Supplementary Materials

The following supporting information can be downloaded at https://www.mdpi.com/article/10.3390/antibiotics13111080/s1: Figure S1: ^1^H NMR and ^13^C NMR spectrum of 2,4-DTBP; Figure S2: ^1^H NMR and ^13^C NMR spectrum of 3,5-DTBP.
