# Supplementary material for: Identification of 2,4-Di-tert-Butylphenol as an Antimicrobial Agent Against Cutibacterium acnes Bacteria from Rwandan Propolis
Source: Antibiotics (Basel). 2024 Nov 13;13(11):1080. doi: 10.3390/antibiotics13111080 (PMC11591542; doi:10.3390/antibiotics13111080)

# Supplementary materials

## Identification from a Rwandan propolis of 2,4-Ditert-butyl phenol as an antimicrobial agent against *Cutibacterium acnes* bacteria

Florent Rouvier, Lydia Abou, Emmanuel Wafo, Perrine André, Julien Cheyrol, Mohamed-Mohsen Khacef, Claude Nappez, Hubert Lepidi and Jean Michel Brunel

Figure S1: $^{1}H$ NMR and $^{13}C$ NMR spectrum of 2,4-DTBP.

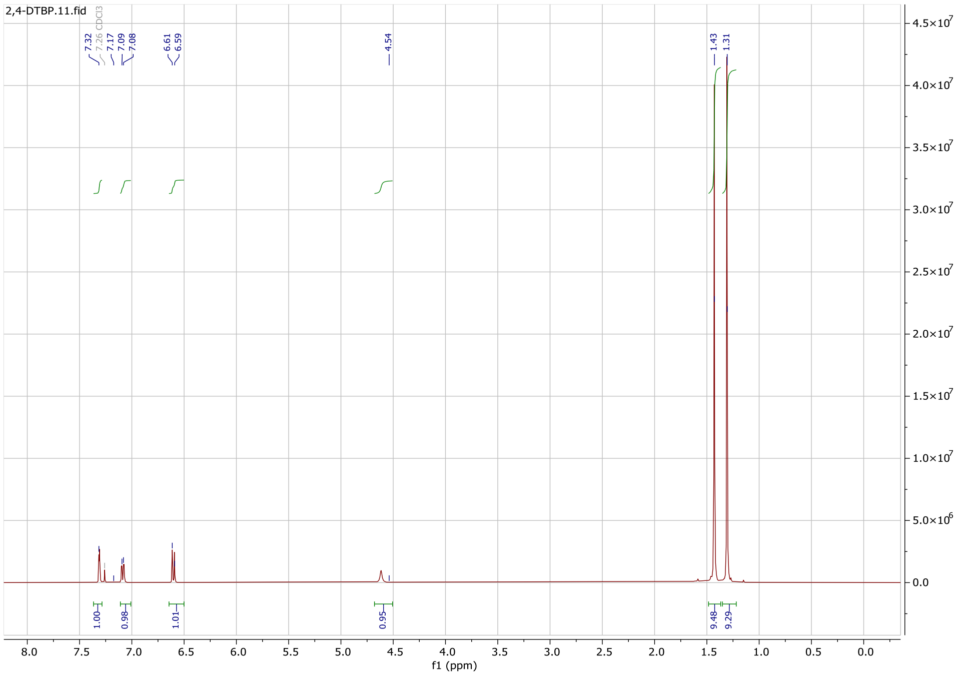


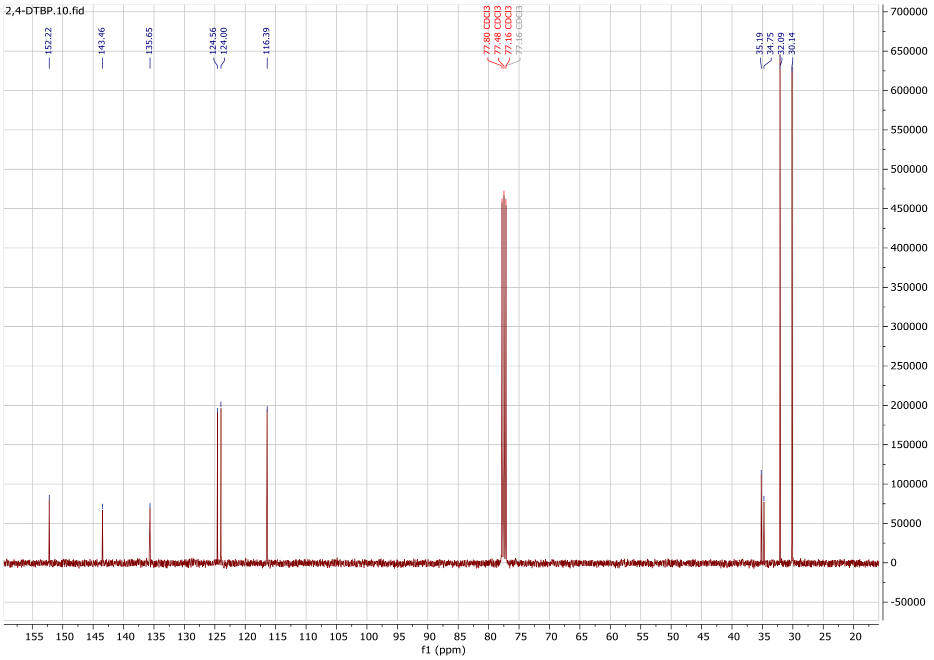

Figure S2: $^{1}H$ NMR and $^{13}C$ NMR spectrum of 3,5-DTBP.

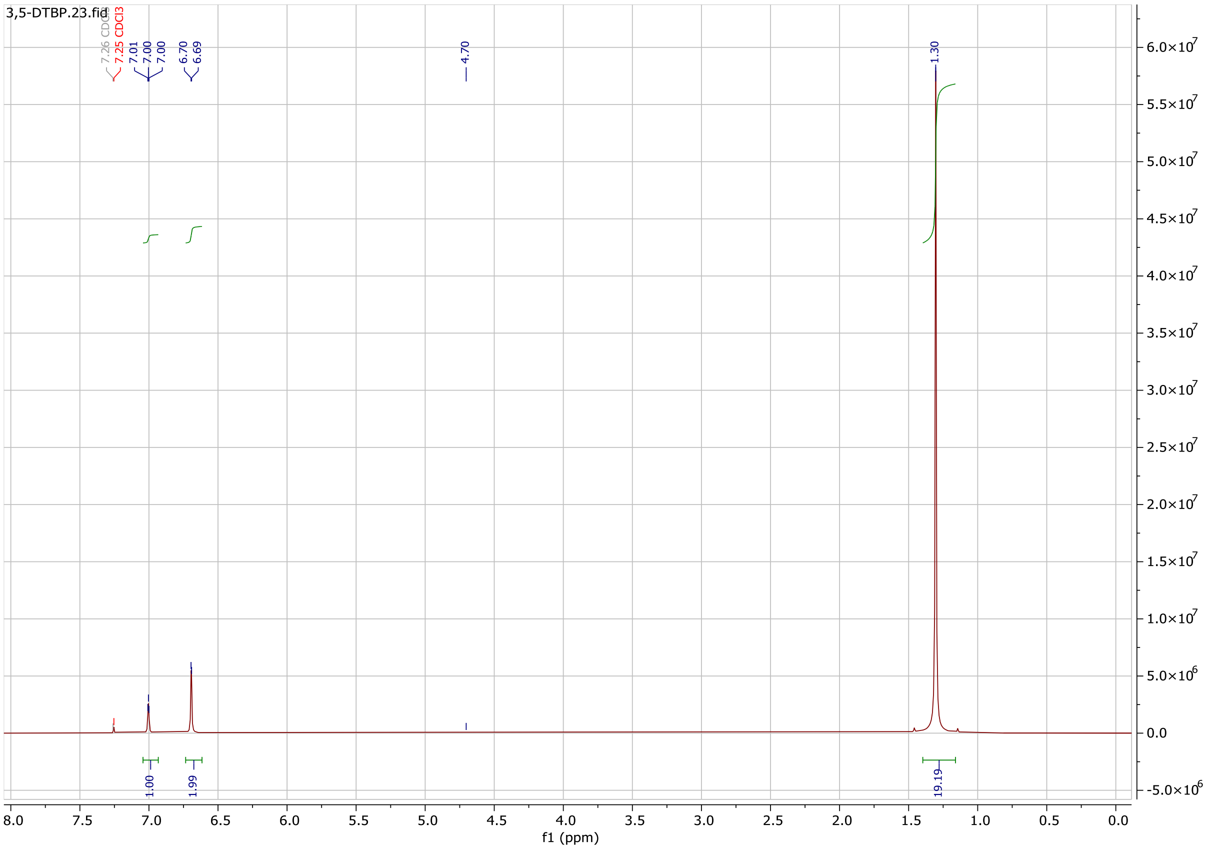


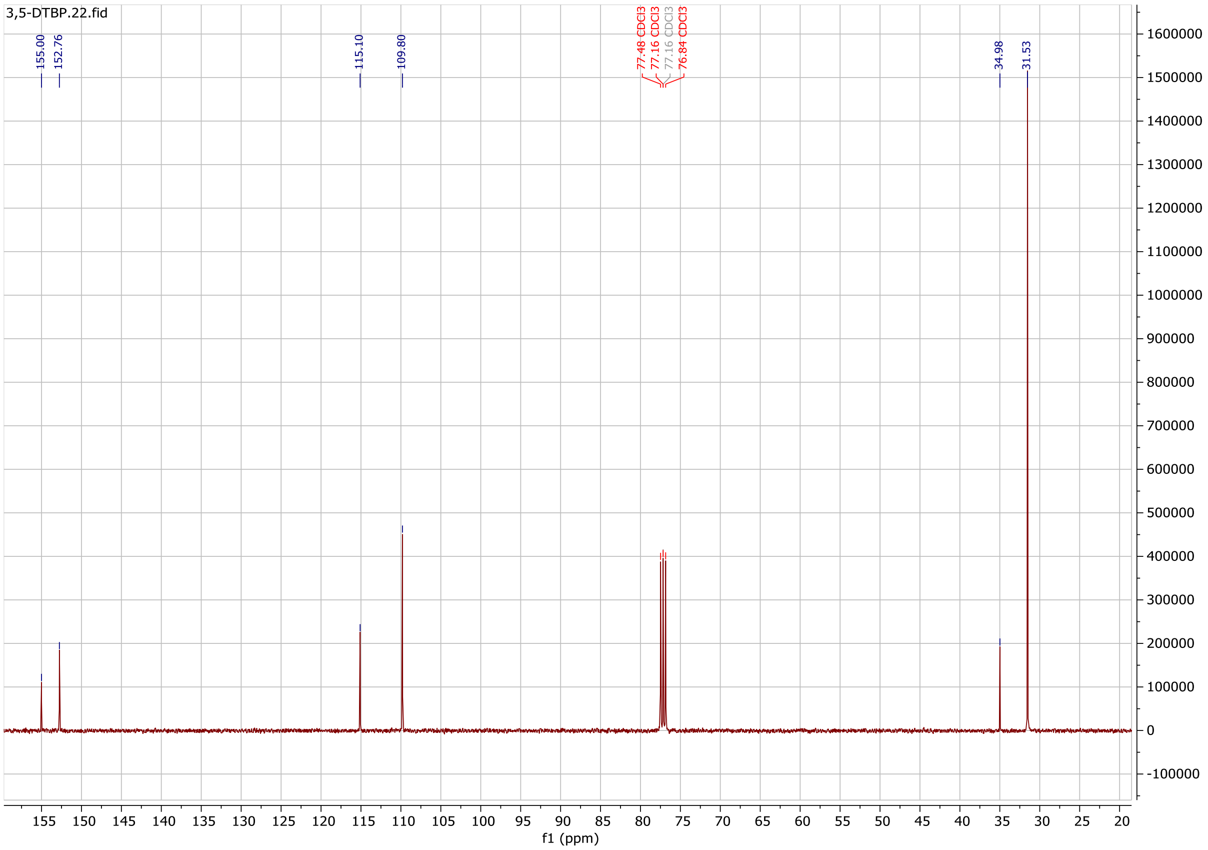

Supplement: Supplementary file 1 [file antibiotics-13-01080-s001.zip › antibiotics-3289070-supplementary.pdf]
